# Regulatory Cues in Pulmonary Fibrosis—With Emphasis on the AIM2 Inflammasome

**DOI:** 10.3390/ijms241310876

**Published:** 2023-06-29

**Authors:** Yu-Hsin Tseng, I-Chen Chen, Wan-Chun Li, Jong-Hau Hsu

**Affiliations:** 1 Department of Pediatrics, Kaohsiung Medical University Hospital, Kaohsiung Medical University, Kaohsiung 80756, Taiwan; 1040809@gap.kmu.edu.tw (Y.-H.T.); yljane@gap.kmu.edu.tw (I.-C.C.); 2 Department of Pediatrics, School of Medicine, College of Medicine, Kaohsiung Medical University, Kaohsiung 80708, Taiwan; 3 Graduate Institute of Medicine, College of Medicine, Kaohsiung Medical University, Kaohsiung 80708, Taiwan; 4 Institute of Oral Biology, College of Dentistry, National Yang Ming Chiao Tung University, Taipei 11221, Taiwan; wcli@nycu.edu.tw

**Keywords:** pulmonary fibrosis, AIM2 inflammasome, epithelial-mesenchymal transition, inflammation, senescence

## Abstract

Pulmonary fibrosis (PF) is a chronic lung disorder characterized by the presence of scarred and thickened lung tissues. Although the Food and Drug Administration approved two antifibrotic drugs, pirfenidone, and nintedanib, that are currently utilized for treating idiopathic PF (IPF), the clinical therapeutic efficacy remains unsatisfactory. It is crucial to develop new drugs or treatment schemes that combine pirfenidone or nintedanib to achieve more effective outcomes for PF patients. Understanding the complex mechanisms underlying PF could potentially facilitate drug discovery. Previous studies have found that the activation of inflammasomes, including nucleotide-binding and oligomerization domain (NOD)-like receptor protein (NLRP)1, NLRP3, NOD-like receptor C4, and absent in melanoma (AIM)2, contributes to lung inflammation and fibrosis. This article aims to summarize the cellular and molecular regulatory cues that contribute to PF with a particular emphasis on the role of AIM2 inflammasome in mediating pathophysiologic events during PF development. The insights gained from this research may pave the way for the development of more effective strategies for the prevention and treatment of PF.

## 1. Introduction

Pulmonary fibrosis (PF), is an end-stage pathological condition characterized by excessive extracellular matrix (ECM) deposition and accumulation due to chronic repetitive alveolar injuries from hereditary factors, infections, and various environmental challenges [1]. PF is classified as an interstitial lung disease (ILD) that commonly leads to the scarring of the lung tissue [2,3]. As the etiology of ILD remained unclear [4], it is believed that fibrosis is a common pathological feature in many chronic inflammatory diseases [5]. Idiopathic pulmonary fibrosis (IPF) is a poor prognostic ILD and has been intensively studied over the past two decades [6]. Most patients with IPF deteriorate within a few years after diagnosis, and acute exacerbation is a major cause of mortality in these patients [7,8]. Although the application of the Food and Drug Administration (FDA) approved two drugs, pirfenidone and nintedanib have shown some benefits in preserving lung function in IPF patients; the therapeutic outcome is still far from satisfactory. To date, lung transplantation is still the only curative therapeutic option for IPF patients [9]. To develop an effective therapeutic scheme is, therefore, urgently needed for IPF patients [10,11]. Inflammasome is a molecular complex of proteins that regulates the secretion of IL-1β and IL-18, both of which are critical for the fibrotic process. In recent years, research on the function of the inflammasome has attracted extensive attention [5,12]. In this review, we emphasize the direct and indirect roles of the AIM2 inflammasome in triggering fibrosis and explore potential novel targets for the treatment of PF.

## 2. Pulmonary Fibrosis

**Figure 1:**
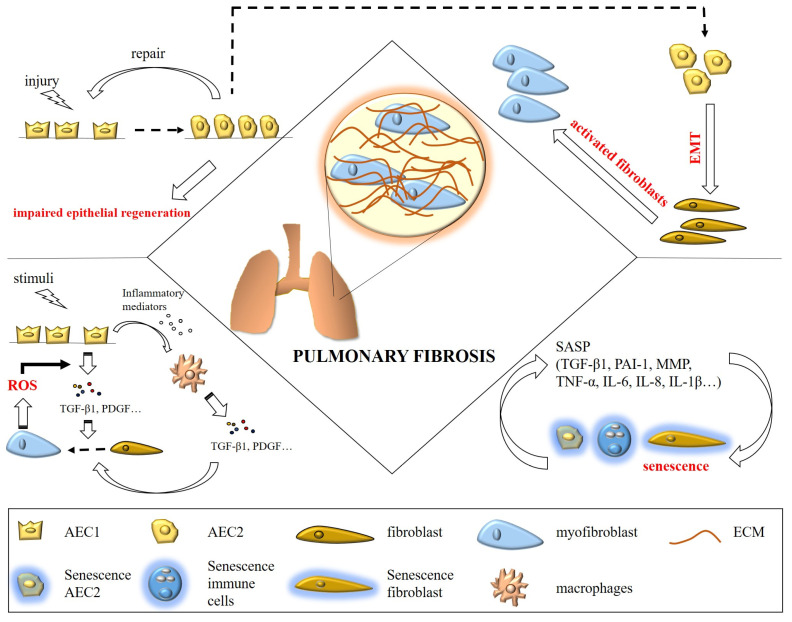
Pathophysiologic mechanisms underlying pulmonary fibrosis are complex and dynamic. These mechanisms include fibroblast activation, epithelial-mesenchymal transition (EMT) of alveolar epithelial cells (AECs), epithelial regeneration impairment, and imbalanced senescence, as well as dysregulation of environmental cues, including immune cell activation deficiency, deregulation of inflammation, and increasing oxidative stress.

Pathologic fibrogenesis is a dynamic process involving complex interactions among cells (e.g., fibroblasts, epithelial cells, and immune cells) and injured endothelium [13]. At the molecular level, progressive fibrosis is associated with fibroblast activation, epithelial-mesenchymal transition (EMT) of alveolar epithelial cells (AECs), lung epithelial regeneration impairment, and imbalanced senescence, as well as dysregulation of environmental cues, including immune cell activation deficiency, deregulation of inflammation, and increasing oxidative stress (Figure 1) [10,14].

### 2.1. Fibroblast Activation

Fibroblasts are the most common cell type in connective tissues and serve to maintain tissue structural integrity by controlling the deposition/degradation of ECM components such as fibronectin and collagen [15]. During both wound repair and pathologic fibrosis, the activated fibroblasts (also called myofibroblasts) expand and become highly mobile, thereby facilitating tissue remodeling through numbers of cell–cell and cell–matrix interactions [15]. The process of fibroblast-to-myofibroblast differentiation can be induced by several factors, such as transforming growth factor (TGF)-β1, connective tissue growth factor (CTGF), platelet-derived growth factor (PDGF), tumor necrosis factor (TNF-α), and interleukin-1 beta (IL-1β) [16,17,18]. In addition, the origin of myofibroblasts is still under debate; as the postembryonic resident lung fibroblasts, the transition of different cells, including fibrocytes, epithelial cells, endothelial cells, adipocytes, monocytes, and mesenchymal cells, may be the potential sources of myofibroblasts [19]. At the cellular basis, myofibroblasts are specialized fibroblasts that acquire the cytoskeletal characteristics of contractile smooth muscle cells and could be distinguished from normal fibroblasts by the detection of upregulated production of α-smooth muscle actin (α-SMA) [20]. It is well acknowledged that myofibroblasts play a central role in the pathogenesis of PF by remodeling ECM [21,22]. In addition to cell-mediated tissue remodeling, myofibroblasts also secrete considerable amounts of growth factors, ECM components, oxidases, and glycosaminoglycans during tissue remodeling in promoting to fibroblast activation and facilitating ECM component deposition. 

### 2.2. Epithelial-Mesenchymal Transition (EMT)

EMT is a potential source of myofibroblasts. Approximately one-third of fibroblasts involved in the development of PF are derived from AECs [23]. In IPF, EMT is regarded as a key process leading to abnormal tissue remodeling [24]. Interestingly, EMT could also be conceptualized as an alternative process of tissue regeneration; thus, factors involved in EMT and mechanisms underlying the disruption of AEC homeostasis could potentially be new diagnostic/prognostic biomarkers or targets of interest for the development of more effective therapeutic schemes for PF [25].

EMT is defined as a biological process in that epithelial cells lose their cell–cell junctions and apical–basal polarity, undergo cytoskeletal remodeling, and acquire mesenchymal features, such as invasion, migration, and ECM production [26]. EMT is a reversible process that occurs not only during embryogenesis but also in response to injury, carcinogenesis, and fibrosis [26,27]. Three major types of EMT have been identified including Type-I EMT in embryogenesis and organ development, Type-II EMT occurring during wound healing, tissue regeneration, and organ fibrosis, and Type-III EMT involved in cancer progression. During organ fibrosis, Type-II EMT serves as a repair machinery in response to persistent inflammation and subsequent organ damage [19]. In experimental settings, EMT is often defined based on various biomarkers. They include (1) alterations of cell–cell contact protein expression (e.g., loss of E-cadherin and gain of N-cadherin); (2) changes of cytoskeletal proteins (e.g., loss of cytokeratins and gain of vimentin, α-SMA, desmin, and fibronectin), and (3) rearrangement of luminal proteins secreted by the original cells (e.g., loss of surfactants and upregulation of ECM components or matrix metalloproteinases (MMPs)) [27,28]. 

Under normal conditions, the expression and/or activation of MMPs and tissue inhibitors of metalloproteinases (TIMPs) regulate ECM homeostasis through an exquisite balance; however, under pathological conditions such as PF, the diverse patterns of secretion and activation of MMPs and their intricated interactions with TIMPs at different locations in vivo are noted. It is interestingly found that MMPs identified in humans play distinct roles in PF pathogenesis. While the impacts of MMP-1, MMP-9, MMP-10, MMP-13, and MMP-14 have not been clearly explored, numbers of profibrotic MMPs, including MMP-2, MMP-3, MMP-8, MMP-11, MMP-12, and MMP-28, are revealed during PF. In contrast, MMP-19 exhibits an antifibrotic role, whereas MMP-7 showed either profibrotic or antifibrotic capacity depending on experimental settings [29]. TIMPs, a four-member family (TIMP 1–4), are endogenous MMP inhibitors and are often abundantly expressed in PF. In general, TIMPs inhibit all MMPs with different suppressive dynamics. For example, TIMP-1 is a strong inhibitor of many MMPs but fails to inhibit MT1-MMP. *Timp1*^−/−^ mice do not differ in terms of fibrosis induction, despite increased inflammation after lung injury being detected [30]. In bleomycin-induced PF, a severe fibrotic response was detected in *Timp3*^−/−^ mice due to persistent inflammation caused by increased neutrophil influx [31]. Moreover, TIMP-3 is strongly stimulated by TGF-β1 in fibroblasts [32], making it speculated if TIMP-3 has an antifibrotic role during PF. Histological studies of patients with IPF have shown that TIMP-1 is present in interstitial macrophages; TIMP-2 is present in fibroblast foci; TIMP-3 is present in the elastic lamina in vessels; and TIMP-4 is present in epithelial and plasma cells, supporting an idea that reduced collagen degradation is the basis of this disorder [33]. Although the roles and interactions of MMPs and TIMPs still required further exploration, they surely play a significant role in regulating PF pathology.

EMT can be regulated by various extracellular ligands, such asTGF-β1, epidermal growth factor, fibroblast growth factor, hepatocyte growth factor, interleukin (IL)-1, connective tissue growth factor, insulin-like growth factor-2, nuclear factor-kB, and Wnt. Most of these regulators could induce intracellular signaling cascades by binding to cell-surface receptors [19,28]. Among them, TGF-β1 is the most common inducer used to activate EMT experimentally. The TGF-β family comprises TGF-β1, TGF-β2, and TGF-β3. Among them, TGF-β1 is dominantly involved in the pathology of PF [34]. TGF-β1 is secreted and stored primarily in ECM, and TGF-β1 signals could be initiated by the binding of ligands and their corresponding receptors, followed by the activation of downstream Smad2 and Smad3 protein through C-terminal phosphorylation. Activation of Smad2 and Smad3 could then bind to Smad4 and form trimers and subsequently translocate into the nucleus, where the Smad complex could regulate target gene expression to exert numbers of biological functions, such as cell proliferation, survival, apoptosis, dormancy, autophagy, and senescence [35,36,37]. It was found that the Smad nuclear complex could inhibit the expression of E-cadherin through SNAIL1 and SNAIL2 transcription factors, thereby inducing the expression of mesenchymal proteins including N-cadherin, fibronectin, and metalloproteinases [28,38]. The TGF-β1/Smad signaling pathway could also indirectly promote EMT transcriptional activity by inducing the expression of *TWIST*, *ZEB1*, and *ZEB2*, as well as the Wnt signaling pathway [37,39]. Moreover, Smad-independent pathways implicated in TGF-β1-dependent EMT include the RhoA, Ras, mitogen-activated protein kinase, phosphoinositide-3-kinase (PI3K), Notch, and Wnt signals. Whilst Smad-dependent and Smad-independent pathways exhibit considerable crosstalk, targeting a single pathway to suppress an EMT-mediated pathological condition is challenging [40,41]. In AECs, TGF-β1 promotes EMT by inducing EMT-related transcriptional repressors (e.g., SNAI1/SNAI2 and ZEB1/ZEB2) and inhibiting NKX2-1, a homeodomain transcription factor involved in the differentiation of lung epithelial cells [42]. M2 macrophages could promote EMT through the TGF-β1/Smad signaling pathway [43]. Various antifibrotic drugs have exerted their therapeutic efficacy by targeting TGF-β1 activity [44,45]. However, direct inhibition of TGF-β1 may lead to various adverse effects due to its pleiotropic effects, making it recommended to target downstream effectors of TGF-β1 signaling for the treatment of PF [36].

### 2.3. Epithelial Regeneration Impairment

The damage of AECs and the subsequential impaired epithelial regeneration are also considered key inducers in the development of PF [46]. The alveolar epithelium comprises type I AECs (AEC1s) and type II AECs (AEC2s). In a normal condition, AEC1s reside over the alveolar surface. However, AEC1s become vulnerable to damage and even death in the presence of lung injury. Upon lung damage occurring, AEC2s, potent alveolar epithelial stem cells, could be activated for alveolar epithelial repair [47]. At the cellular and molecular basis, lung injury could induce the proliferation of surfactant-producing AEC2s to form wound clots that cover alveolar surfaces, activate local coagulation pathways, and enhance the formation of a provisional matrix [11,48]. During nonpathological wound healing, hyperplastic AEC2s could transdifferentiate to AEC1s followed by gradual degradation of provisional matrix, allowing lung tissues to restore their structure and function [49,50]. It was found that cells in the intermediate state of AEC2-to-AEC1 transdifferentiation, called new transition state cells, may regenerate the alveolar epithelial surface and facilitate repair [49]. During the postinjury transdifferentiation, AEC2s fail to convert into AEC1s, resulting in insufficient repair, which leads to subsequent PF [51,52]. In other words, persistent disturbance of the epithelial basement membrane may cause an abnormal wound repair that could be attributed to the inability of AEC2s to transdifferentiate into AEC1s. The unsuccessful AEC2s-to-AEC1s transdifferentiation could result in endoplasmic-reticulum stress, defective autophagy, mitochondrial dysfunction, apoptosis, inflammatory cell recruitment, profibrotic signaling, and altered progenitor function, ultimately converging to drive downstream fibrotic remodeling [53]. 

### 2.4. Imbalanced Senescence

The defect of senescence of AECs, fibroblasts, and immune cells may promote PF [54]. Cellular senescence, defined as a state in which aged or damaged cells fail to proliferate despite the presence of optimal growth conditions, is a programmed aging process. Senescence involves the irreversible cell cycle arrest through the induction of cyclin-dependent kinase inhibitors and lysosomal enzymes, thereby leading to the onset of the senescence-associated secretory phenotype (SASP) [55]. SASP is comprised of different factors, such as TGF-β1, and secreted inflammatory factors, such as plasminogen activator inhibitor-1, MMPs, TNF-α, interleukin (IL)-6, IL-8, and IL-1β. During wound healing, SASP plays an important physiologic role in cell proliferation and differentiation; on the other hand, the deregulated expression of these factors could contribute to the pathophysiology of PF. It was found that SASP molecules were present in the IPF lungs and allowed senescent cells to contagiously spread the senescence phenotype from one cell type to another and eventually lead to a systemic cellular dysfunction [56].

At the cellular level, the senescent of AEC2s is a characteristic of lung epithelial injury in IPF. Senescent AEC2s secreted SASP and profibrotic factors, particularly TGF-β1 to induce the activation of myofibroblasts and to promote the ECM deposition, resulting in an irreversible fibrotic damage [57]. Another study also showed that senescent fibroblasts in IPF subjects suppressed epithelial-cell proliferation to inhibit wound repair and secreted high levels of proinflammatory cytokines/chemokines, profibrotic factors, and ROS, affecting themselves or neighboring cells through autocrine and paracrine effect, respectively [58]. In the context of senescence, the immune system can be affected in quantity (dead in immune cells) and quality (dysfunctional, so-called immunosenescence) in PF patients [59]. Under normal physiologic conditions, senescent cells could activate both innate and adaptive immune responses to maintain tissue and organ homeostasis; nevertheless, the persistence and accumulation of senescent cells may be involved in the pathophysiology of aging and age-related diseases, including PF [56,60]. Immunosenescence includes innate immunosenescence, which is primarily detected in alveolar macrophages, natural-killer (NK) cells, and dendritic cells (DCs), and adaptive immunosenescence, which is mainly driven by the shift of B and T cells toward a memory state [61]. For example, in the bronchoalveolar lavage and lungs of IPF patients, the dysregulation of innate immunity increases the numbers of immature DCs, which are a source of several proinflammatory cytokines in autoimmunity. At the end-stage IPF, the reduced amount of mature CD57^+^ cytotoxic NK cells and leukocytes is associated with defective immunosurveillance for the clearance of senescent cells, which results in a profibrotic microenvironment [62]. It was also found that the increase in the number of regulatory T cells during aging promoted the differentiation of Th17 cells and the production of IL-17 in experimental PF. IL-17A, secreted by helper T cells, contributed to rheumatoid arthritis-induced PF through the IL-17A/IL-17RA regulatory axis [63].

### 2.5. Immune-Cell Activation Deficiency

Abnormalities of innate and adaptive immunity contribute to fibrosis but the role of adaptive immunity is less clear. Among innate immune cells, macrophages are considered to be the most important cells in fibrotic lung disease [64]. It was previously reported that alveolar macrophages are highly plastic in inflammatory status, exerting either pro- or antifibrotic roles according to their polarization, local microenvironment, and disease stage [65]. During tissue injury and early inflammation, an active cytokine environment drives the activation of proinflammatory M1 macrophages to promote inflammation and eliminate pathogens. By contrast, the activation of anti-inflammatory/profibrotic M2 macrophages is associated with the resolution of acute inflammation [66]. M2-like macrophages promote PF by producing profibrotic cytokines (e.g., TGF-β1 and platelet-derived growth factor), fibronectin, and TIMPs. Inhibition of M2 macrophage polarization suppresses EMT, impedes myofibroblast differentiation, and reduces TGF-β1–Smad signaling, thereby decelerating the development of PF [64,67,68]. Macrophage-associated regulatory cues for PF include TGF-β1/Smad, Wnt/β-catenin, and IL signaling pathways [43]. Although the roles of M1 and M2 macrophages in the process of PF are complex and multimachineries yet to be elucidated, it was indeed found that an unbalanced M1/M2 polarization contributes to the exacerbation of the typical pathogenesis in PF patients [69].

Moreover, neutrophils play regulatory roles in both innate and adaptive immune response and might modulate fibrogenesis in the lung [70,71]. In the early inflammatory stage, the infiltrating neutrophils may secrete proinflammatory cytokines such as IL-1β, TNF-α, IL-6, and IL-8 to injure the lung tissue. On the contrary, neutrophils could produce profibrotic mediators in the chronic fibrotic stage [72]. It is interestingly found that the numbers of neutrophils in the lungs and sputum, as well as levels of IL-8 (a chemokine attracting neutrophils to sites of inflammation) in bronchoalveolar lavage fluid (BALF), are higher in IPF patients than that from healthy donors. Increased neutrophil percentage in BALF taken from patients with IPF is considered an independent predictor of early mortality [73]. On the other hand, neutrophils were also considered to participate in the pathogenesis of IPF by controlling the balance of TIMPs and MMPs (such as the profibrotic MMP-2, MMP-8, and MMP-9) [74]. Neutrophil-derived leukotrienes have been suggested to stimulate the infiltration of other immune cells into the lung, thereby promoting the progression of IPF. Additionally, abnormally high serum levels of neutrophil elastase (NE) have been found in patients with acute exacerbation of IPF [72]. NE is involved in ECM turnover as well as promotes the proliferation of lung fibroblasts and myofibroblast differentiation in lung fibrosis [70,75]. NE-deficient mice are free of fibrosis in several models, while Sivelestat, a potent NE inhibitor, could inhibit fibrosis in the bleomycin model [76].

### 2.6. Inflammation and Oxidative Stress

Deregulated inflammation and oxidative stress are closely associated and played an important role in facilitating PF development [41]. Lung tissues are exposed to a higher level of oxygen compared to other tissues, making the cellular redox state and the oxidant–antioxidant imbalance could be key determinants for PF development in both human and mouse models of IPF [77]. In the pathogenesis of PF, alveolar macrophages play a critical role by initiating an immune response and generating reactive oxygen species (ROS), particularly mitochondrial H_2_O_2_. Abrogation of mitochondrial oxidative stress limits the activation of the inflammasome and attenuates the development of PF in mice [78,79]. As it is widely appreciated that the regulation of ROS is essential for redox imbalance caused by exogenous (e.g., hyperoxia, cigarette smoke, asbestos fibers, drugs, radiation, and pathogens) or endogenous (e.g., superoxide radical, hydrogen peroxide, and hydroxyl radical) stimuli, ROS may exert extensive effects on various cells such as epithelial cells, myofibroblasts, and inflammatory cells, as well as the production of growth factors (e.g., TGF-β1), proteases (e.g., MMPs), protease inhibitors, and ECM components, ultimately resulting in the development of end-stage fibrosis [80]. It has been reported that TGF-β1-activated nicotinamide adenine dinucleotide phosphate oxidase in human fibroblasts, leading to an increased ROS production [81,82]. Interestingly, ROS, in turn, increased the release of TGF-β1 from pulmonary epithelial cells and could directly activate TGF-β1 by disrupting its interaction with latency-associated peptide, revealing a positive feedback regulation in the myofibroblast microenvironment [83,84]. 

## 3. Inflammasome

Inflammasomes are critical components of the innate immune system that could be activated in response to various stimuli and the activation of inflammasomes is found to induce PF [10,85,86,87]. In 2002, the term “inflammasome” was first coined by Martinon et al. to describe the presence of an NLRP1 complex activated by caspase-1/caspase-5 in leukocytes treated with inflammatory stimuli. Later, six widely accepted canonical inflammasome complexes were identified, including the nucleotide-binding and oligomerization domain (NOD)-like receptor protein (NLRP)1 inflammasome, the NLRP3 inflammasome, the NOD-like receptor (NLRC)4 inflammasome, the absent in melanoma (AIM)2 inflammasome, the IFI16 inflammasome, and the Pyrin inflammasome [88]. Inflammasomes are ubiquitously detected in the cytoplasm of cells, including immune cells (e.g., monocytes, macrophages, B cells, T cells, and dendritic cells) and nonimmune cells (e.g., hepatic stellate cells, fibroblasts/myofibroblasts, epithelial cells, endothelial cells, and parenchymal cells) [5,89,90,91]. Based on the activation of different caspases in the inflammatory pathway, inflammasomes could be classified into canonical or noncanonical inflammasomes. During canonical inflammasome activation, endogenous or exogenous stimulations on backbone proteins could trigger inflammasome assembly and subsequently activate procaspase-1 to generate mature caspase-1. Then, mature caspase-1 cleaves its cytokine substrates, such as IL-1β and IL-18, into mature cytokines, which are next secreted outside the cells. Noncanonical inflammasomes, unlike canonical inflammasomes that activate caspase-1, mainly activate caspases other than caspase-1 (e.g., caspase-4, caspase-5, and caspase-11) [92,93]. 

Over the past years, there have been significant studies focusing on the roles of N LRP1, NLRP3, NLRC4, and AIM2 inflammasomes in controlling pathological conditions [5]. Among them, activation of NLRP3 or AIM2 inflammasome-dependent inflammation is thought to play an important role in the progression of PF [87]. The NLRP3 inflammasome complex formation and activation is induced by various bacterial and viral particles, while the AIM2 inflammasome is activated by double-strand DNA (dsDNA) derived from bacteria, viruses, and abnormal hosts [87]. NLRP3 consists of an amino-terminal pyrin domain (PYD), a central nucleotide-binding and oligomerization domain (NOD), and a C-terminal leucine-rich repeat (LRR) domain. The PYD domain of NLRP3 acts as a receptor to interact with the pyrin domain of the adaptor protein ASC to initiate inflammasome assembly. The NOD domain exhibits ATPase activity that is vital for NLRP3 oligomerization and has been implicated as the target of NLRP3 inhibitors. However, the evolution and function of the LRR domain is not well understood [94]. It is known that the NLRP3 inflammasome is overactivated in IPF patients, leading to increased production of ECM components [95,96]. In several animal models, studies have demonstrated the involvement of NLRP3 inflammasome activation in the mechanisms underlying PF. Moreover, the therapeutic impacts of potent NLRP3 inflammasome inhibitors, such as OLT1177, Tranilast, Oridonin, CY-09, and MCC950, were demonstrated in cells and animal models of diabetes, acute arthritis, and Parkinson’s disease [97,98,99,100,101,102]. 

Unlike the NLRP3 inflammasome, the AIM2 inflammasome inhibitors remain uncovered and are not identified as therapeutic agents for human disease [87]. In this review article, we aim to elaborate on the molecular machinery underlying PF, in particular, to emphasize the role of the AIM2 inflammasome in controlling PF, providing possible insights into AIM2-mediated therapy for this disease.

### 3.1. AIM2 Inflammasome

**Figure 2:**
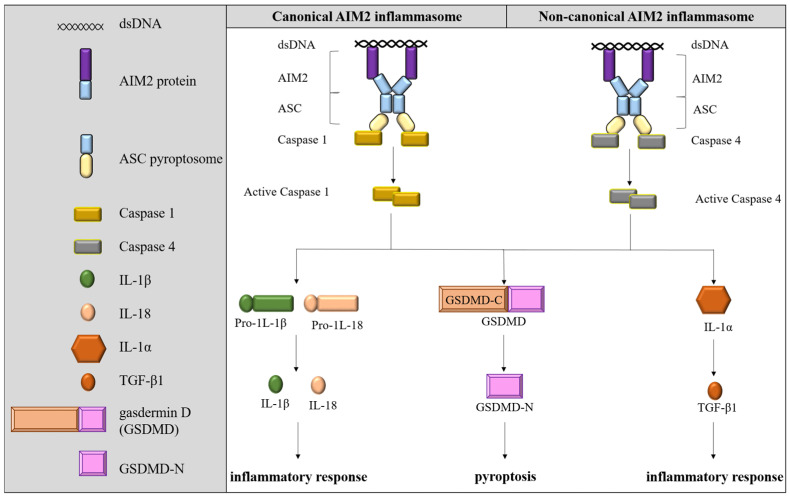
AIM2 inflammasomes are classified into canonical AIM2 inflammasomes and noncanonical AIM2 inflammasomes. Canonical AIM2 inflammasomes mainly initiate caspase-1 activation by interacting with cytokine substrates (e.g., IL-1β and IL-18) to form mature cytokines, which are subsequently secreted extracellularly to induce inflammatory responses. Noncanonical AIM2 inflammasomes activate noncaspase-1 caspases (e.g., caspase-4). AIM2 activation will finally lead to pyroptotic cell death.

AIM2 was originally discovered as a γ-interferon-inducible protein [103]. In a later study, AIM2 is redefined as a nucleic acid sensor that recognizes aberrant cytoplasmic double-stranded DNA (dsDNA) in cells through its oligosaccharide domain [104,105]. AIM2 consists of an N-terminal pyrin domain and a C-terminal HIN-200 domain [106]. Once AIM2 binds with cytoplasmic dsDNA, the complex next binds to apoptosis-associated speck-like protein containing a CARD (ASC) through the AIM2 pyrin domain, thus forming the AIM2 inflammasome [104,107,108]. Canonical AIM2 inflammatory complex activates caspase-1 through an autoproteolysis process and activated caspase-1 converts pro-IL-18 and pro-IL-1β into their active forms for cell lytic death called pyroptosis by cleaving gasdermin D (GSDMD) [104,109]. AIM2 is critical for inflammasome activation in response to *Francisella tularensis*, vaccinia virus, and mouse cytomegalovirus, as well as also playing a minor role in the sensing of Listeria monocytogenes infection. *Aim2* null macrophages are functionally impaired based on their reduced ability to release IL-1β [110]. In a mouse model of unilateral urethral obstruction, *Aim2* knockout led to defective renal injury, fibrosis, and inflammation compared with control subjects [111]. In contrast, noncanonical AIM2 inflammasome activates distinct caspase for related downstream physiological regulations. For example, cytosolic lipopolysaccharide (LPS) from Gram-negative bacteria and their outer membrane vesicles could directly trigger caspase-11 and caspase-4 activation, further cleaving GSDMD to induce pyroptosis. During pyroptosis, cells could release IL-1β and IL-18 cytokines into the extracellular space, thereby inducing a severe inflammatory response via an activation of the IL-1R/IL-18R-MyD88-NFκB regulatory axis [109,112]. Figure 2 illustrates the activation of the AIM2 inflammasome.

### 3.2. Role of the AIM2 Inflammasome in Pulmonary Fibrosis

AIM2 inflammasome-mediated pyroptosis, in both macrophages and epithelial cells, is involved in the development of radiation-induced tissue injury [113,114]. The upregulation of AIM2 expression has been observed in alveolar macrophages from patients with IPF and in a mouse model of PF induced by bleomycin [87,115]. In PF, fibrotic lung remodeling is driven by the TGF-β1-mediated inflammatory response, where TGF-β induction was through an AIM2-activated caspase-4 dependent manner (noncanonical inflammasome) but not via a caspase-1- or TLR4-dependent pathway. The AIM2-mediated noncanonical inflammasome activation could induce the release of IL-1α, a cytokine responsible for the release of TGF-β from the peripheral blood mononuclear cells (PBMCs) of patients with IPF [85]. In contrast, in patients with chronic obstructive pulmonary disease (COPD), a chronic inflammatory lung disease, both AIM2-mediated canonical and noncanonical inflammasomes participate the IL-1α-induced release of TGF-β in PBMCs [116]. At the molecular basis, recent studies reported that histone deacetylase 3 (HDAC3) expression is upregulated in animal models of bleomycin-induced PF. HDAC3, an important zinc-dependent metalloenzyme, can affect various disease states in humans through epigenetic modulations. The role of HDAC3 is known to be associated with several life-threatening diseases such as cancer, inflammatory diseases, cardiovascular diseases, neurodegenerative diseases, learning and memory dysfunctions, Huntington’s disease (HD), and diabetes [117]. Moreover, HDAC3 promotes EMT, inflammation, and PF development through activation of the Notch1/signal transducer and activator of transcription 1 (STAT1) signaling pathway and the AIM2 inflammasome [118].

### 3.3. Discovery of AIM2 Inflammasome Inhibitors

As mentioned in the previous section, FDA-approved drugs, pirfenidone, and nintedanib are currently used to treat PF [119]. Even though these two antifibrotic drugs could prevent further scarring and decelerate fibrosis progression in patients with PF, they do not ensure a complete cure for the disease [11]. The activation of the AIM2 inflammasome is associated with pathological conditions of PF and drugs targeting AIM2 inflammasome have been found to be effective for PF treatment. For example, in a radiation-induced lung injury (RILI) model, Re-Du-Ning (RDN), a traditional Chinese medicine composed of *Lonicera japonica* Thunb., *Gardenia jasminoides* Ellis, and *Artemisia annua* L., considerably ameliorated RILI by inhibiting the infiltration of inflammatory cells and by suppressing the expression of proinflammatory cytokines, such as IL-1β, IL-6, and TNF-α. A network pharmacology analysis indicated that RDN could improve radiation-induced pneumonitis via an inhibition of AIM2-mediated pyroptosis. Moreover, RDN may also reduce EMT and PI3K/protein kinase B signaling activity [120]. Another study using a RILI model indicated that the andrographolide could largely ameliorate radiation-induced lung-tissue damage, reduce inflammatory cell infiltration, suppress proinflammatory cytokine release, and improve progressive fibrosis. The group further confirmed that inhibition of radiation-induced activation of the AIM2 inflammasome and pyroptosis may be the underlying machinery for the rescue of lung injury. This conclusion was based on the results showing that in bone-marrow-derived macrophages exposed to 8 Gy of X-ray radiation, andrographolide effectively prevented AIM2 from translocating into the nucleus to sense DNA damage, thereby inhibiting AIM2 inflammasome-mediated pyroptosis [114]. In a bleomycin-induced PF model, a previous study found that RGFP966, a specific HDAC3 inhibitor, ameliorated bleomycin-induced PF through regulations of EMT and reduced expression of the proinflammatory cytokines IL-1β and IL-18. Furthermore, RGFP966 downregulated the Notch1 and STAT1 signaling activity and AIM2 inflammasomes both in bleomycin-induced PF mice in vivo and TGF-β1-stimulated MRC-5 cells in vitro. The molecular analysis uncovered that HDAC3 contributes to the development of PF through Notch1, STAT1, and AIM2 activity [118]. Anti-inflammatory and antifibrotic drugs modulating AIM2 activity in pulmonary fibrosis are summarized in Table 1.

**Figure 3:**
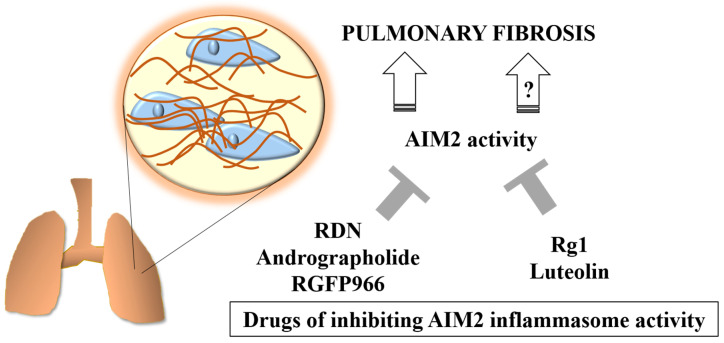
Drugs inhibiting AIM2 inflammasome activity. RDN, andrographolide, and RGFP966 can inhibit pulmonary fibrosis by impairing the activity of the AIM2 inflammasome. Rg1 and luteolin inhibit the AIM2 inflammasome in lipopolysaccharide-stimulated BV-2 cells and NSCLC xenograft mouse models, respectively. The question mark represents whether Rg1 and luteolin have the potential to treat pulmonary fibrosis is unclear.

Other than PF models, inhibition of the AIM2 inflammasome has also been applied in neuroinflammation and cancer treatment. For instance, the activation of inflammasomes is associated with neuroinflammation. Ginsenoside Rg1 (Rg1) is a major component of ginseng; its neuroprotective effects associated with its anti-inflammatory and antioxidant properties have been widely recognized [121,122]. Rg1 effectively inhibits inflammasome activation and cell pyroptosis in lipopolysaccharide-stimulated BV-2 cells. The analysis of drug prediction using Rg1-related data from the SwissTargetPrediction database revealed that STAT3 has the highest threshold of probability score, suggesting Rg1 can regulate STAT3 phosphorylation, which binds to the promoter region of AIM2 [123]. Furthermore, AIM2 is overexpressed and exhibits oncogenic properties in oral squamous-cell carcinoma [124] and nonsmall-cell lung cancer (NSCLC) [125]. Luteolin, a natural flavonoid with anti-inflammatory properties, is one of the key mechanisms underlying its pharmacologic effects. The antitumor effects of luteolin on NSCLC, including the induction of G2/M phase arrest and inhibition of EMT, are dependent on AIM2. Therefore, to suppress AIM2 may be a therapeutic strategy for NSCLC [126]. To the best of our knowledge, Rg1, and luteolin have not been studied in PF, making them attractive for future studies to test their therapeutic roles in PF. Collectively, Figure 3 presents drugs that could inhibit AIM2 inflammasome activity.

## 4. Conclusions

Fibrotic diseases account for up to 45% of deaths in developed countries, revealing the urgency of preventing and treating PF [127]. Although pirfenidone and nintedanib approved by the Food and Drug Administration are currently used for IPF therapy, there is still much room for improvement in clinical efficacy. The evidence suggests that activation of the inflammasome, especially the NLRP3 and AIM2 inflammasomes, contributes to pulmonary fibrosis; however, the molecular mechanisms associated with the AIM2 inflammasome and the utility of AIM2 inflammasome inhibitors as therapeutic agents in PF remain to be investigated. We elucidated the pathologic mechanisms underlying PF, with a specific emphasis on the role of the AIM2 inflammasome. Moreover, we summarized the potential drug candidates capable of inhibiting AIM2 inflammasome activity. We aim to facilitate the discovery of and to develop novel effective drugs for PF in the future.

## Figures and Tables

**Table 1:** Anti-inflammatory and antifibrotic drugs modulating AIM2 activity in pulmonary fibrosis.

Drug	Pulmonary Fibrosis Model	Effect	Ref
RDN	(1) RILI in Female C57BL/6 mice	🡲 Ameliorates pneumonitis 🡲 Inhibits cell pyroptosis 🡲 Inhibits EMT and PI3K/AKT pathway	[120]
ANDRO	(1) RILI in Female C57BL/6 mice (1) 8 Gy of X-ray radiation-stimulated BMDMs	🡲 Ameliorates lung tissue damage, inflammatory cell infiltration, and proinflammatory cytokine release 🡲 Inhibits progressive fibrosis 🡲 Ameliorates RILI	[114]
RGFP966	(1) A bleomycin-induced pulmonary fibrosis mouse model (2) TGF-β1-challenged MRC-5 cells	🡲 Ameliorates pulmonary fibrosis 🡲 Inhibits fibrotic phenotypes	[118]

RDN: Re-Du-Ning; ANDRO: andrographolide; RILI: radiation-induced lung injury; EMT: epithelial-mesenchymal transition.

## Data Availability

Not applicable.
